# Comorbidity between autism spectrum disorder and obsessive compulsive disorder. A case series

**DOI:** 10.1192/j.eurpsy.2025.1709

**Published:** 2025-08-26

**Authors:** P. Del Sol, L. Mallol, A. Izquierdo, R. Fernández, M. García Moreno

**Affiliations:** 1 Hospital Puerta de Hierro, Madrid; 2 Hospital Infanta Cristina, Parla, Spain

## Abstract

**Introduction:**

Autism spectrum disorder is a complex pathology that can occur very frequently with other disorders. It is known to co-occur with ADHD (attention deficit hyperactivity disorder), anxiety disorders or depression. Comorbidity with obsessive-compulsive disorder (OCD) can be difficult to detect since rigid and obsessive thinking, which in many cases is similar to the obsessions and compulsions of the OCD patient, is part of the core symptomatology of autism spectrum disorder.

**Objectives:**

To present two cases of patients atteded in the Adolescent Hospitalization Unit with Autism Spectrum Disorder comorbid with OCD

**Methods:**

Two clinical cases are presented, with review of literature.

**Results:**

**First case**

This is a 16-year-old male, diagnosed with autism spectrum disorder at age 4. He begins with intense preoccupation for hygiene, developing cleaning rituals. He begins to develop the idea that his cells are released from his body and that they are sensitive to external stimuli in the same way that he is. He claims that in order for them to be eliminated and “not to suffer” he has to wash them and make flapping movements and avoid their accumulation.

He begins to treat with sertraline up to 200 mg with partial response, adding fluvoxamine 100 mg with important improvement of the symptomatology.

**Second case**

17-year-old male admitted to the inpatient unit for isolation and cleaning rituals and fear of contamination. The patient had no past psychiatric history. He had been out of school for 4 months. The patient began to develop a fear of pathogen contamination. He maintained cleaning and checking rituals. He ate only packaged food and spent hours, and slept, in the same corner of the bed.

Medication with sertraline up to 150 mg was started with progressive improvement. During the admission a history of evolutionary development was taken, observing a difficulty in the mentalization of other people’s emotions. Very adult and literal speech with difficulty in abstract thinking. He revealed presence of restricted interests throughout his history such as rare languages. Finally, the diagnosis of Autism Spectrum Disorder and comorbid obsessive compulsive disorder was made.

**Conclusions:**

The comorbidity between autism spectrum disorder and OCD occurs between 37%. This comorbidity can make it difficult to perform psychotherapeutic interventions. This work shows on the one hand an already diagnosed autistic patient who develops an OCD, and on the other hand, how the initial diagnosis is an OCD on which an autism spectrum disorder is detected. This highlights the importance of knowing the comorbidity in order to detect both diagnoses.

**Disclosure of Interest:**

None Declared

